# Synthesis and characterization of piperazine-substituted dihydrofuran derivatives viaMn(OAc) _3_ mediated radical cyclizations

**DOI:** 10.3906/kim-2003-23

**Published:** 2020-10-26

**Authors:** Sait SARI, Mehmet YILMAZ

**Affiliations:** 1 Department of Chemistry, Faculty of Arts and Sciences, Kocaeli University, 41380 Umuttepe, Kocaeli Turkey

**Keywords:** Piperazine, dihydrofuran, Mn(OAc) _3_, radical cyclization

## Abstract

The aim of this study is to synthesize novel piperazine-containing dihydrofuran compounds (3a-n)from radical additions and cyclizations of diacyl and alkyl-acyl piperazine derivatives (1a-h) with 1,3-dicarbonyl compounds (2a-c) mediated by Mn(OAc) _3_ for the first time. From the reactions of 1a-c with dimedone (2a);1a, 1c, and 1d with acetylacetone (2b); and 1a with ethylacetoacetate(2c) ,the dihydrofuran-piperazine compounds 3a-c, 3d-f, and 3g were obtained in medium to high yields (31%–81%), respectively. In addition, dihydrofuran-piperazine compounds 3h-j and 3k-n were prepared at low to medium yields (20%–40%) from the reactions of **1e-g** with **2a** and **1e-h** with **2c** , respectively.

## 1. Introduction

Heterocycles are important compounds and have gathered much attention due to their biological properties, and many synthetic drugs contain heterocyclic scaffolds [1,2]. Piperazine is considered a privileged scaffold in medicinal chemistry [3], and there are many biological activity studies in the literature for piperazine-bearing compounds such as antibacterial [4], anticonvulsant [5], antituberculosis [6], antiviral [7], anticancer [8], and acetylcholinesterase inhibition [9,10]. Piperazinecan be found in active drug ingredients such as imatinib [11], sildenafil [12], indinavir [13], and gatifloxacin[14]. In addition, there have been anticonvulsant activity [15], monacylglyserine lipase inhibition [16], antimicrobial [17], and antiinflammatory activity studies [18]for cinnamylpiperazines and antimycobacterial [19], antiischemic [20], and antiparasitic activity studies [21] for acrylamide piperazine derivatives.

Dihydrofurans have gathered much attention due to their biological activities and have great potential as building blocks for pharmaceutical agents. Sarcophytoxide [22], clerodin [23], fercoprolone [24], and austocystin [25] are natural bioactive compounds that carry dihydrofuran moieties. Dihydrofurans can be obtained via transition metal salts which are capable of transferring single electrons (Mn ^3+^ , Ce ^4+^ , Co ^3+^ , etc.) to active methylene compounds to form α-carbon radicals. The addition of these radicals to unsaturated systems is used to generate new C-C bonds [26–28]. Manganese (III) acetate [29–33] and cerium(IV) ammonium nitrate (CAN) [34–38] are widely used in these reactions. Our research group has reported radical addition and cyclization reactions with CAN [39–42] and radical cyclization reactions of 1,3-dicarbonyl derivatives with various unsaturated systems, such as conjugated amide derivatives [43–47]and heteroaromatic conjugated alkenes [48–51].

In this work we report new dihydrofuran-containing piperazine compounds (3a-n) viaMn(OAc) _3_ mediated radical cyclization in medium to high yields. All new compounds were characterized by ^1^ HNMR, ^13^ C NMR, HRMS, and FTIR spectroscopy.

## 2. Results and discussion

In our previous work [52] diacyl and alkyl-acylpiperazine derivatives were obtained; in this work these compounds (1a-h) were used as starting reagents to synthesize piperazine-containing dihydrofuran molecules.

Novel piperazine–dihydrofuran compounds (3a-n) were synthesized via Mn(OAc) _3_ mediated oxidative radical cyclization reactions of unsaturated diacyl (1a-d) and alkyl-acyl (1e-h) piperazine derivatives ,as well as 1,3-dicarbonyl compounds such as dimedone (2a), acetylacetone (2b), and ethylacetoacetate (2c). All radical cyclizations were carried out at 1.2:1:2 molar ratios [piperazine derivative:1,3-dicarbonyl:Mn(OAc) _3_ ].

The results of the reactions of 1a-d with 2a-care given in Table 1. The treatment of 1a-c with dimedone (2a) gave dihydrofurans3a (81%), 3b (50%), and 3c (64%), respectively, in moderate-to-good yields. Although compounds 1a and 1b are similar, there is a significant difference in product yields obtained from them (3aand 3b, respectively). The steric hindrance originated through methyl substitution on alkene moiety of 1b caused the relatively low yield of 3b. Compounds 3d (73%), 3e (52%), and 3f (31%) were obtained as a result of reactions between 1a, 1c, and 1d with 2b in moderate-to-good yields, respectively. Through the reaction of 1a with 2c, compound 3g was isolated at a 60% yield. All cyclizations occurred at the aromatic-ring–carrying sides of the piperazines. This is because radical intermediates formed adjacent to aromatic rings have greater stability than those formed adjacent to methacryloyl alpha carbons on carbon atoms (Figure, Intermediate C and F).

**Table 1:** Radical cyclizations of 1a-d with 2a-c.

Entry	Piperazine	1,3-dicarbonyl	Product	Yield (%) ^a^
1	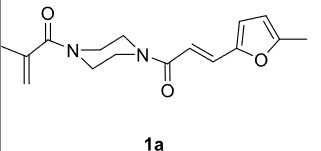	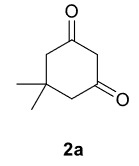	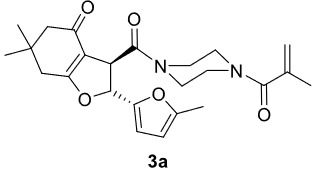	81
2	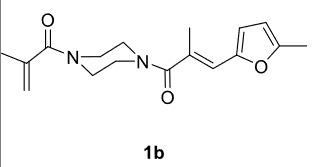	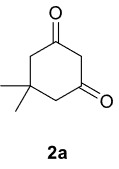	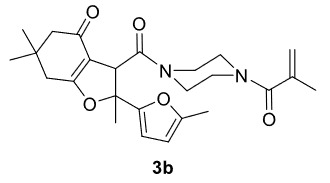	50
3	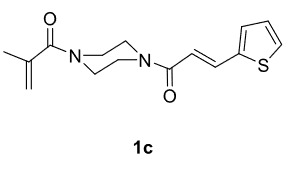	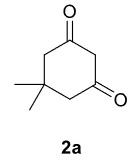	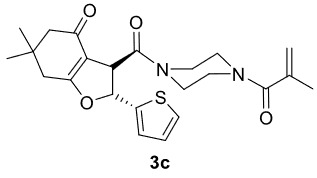	64
4	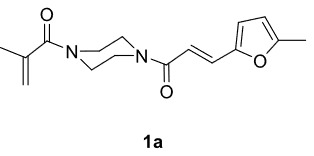	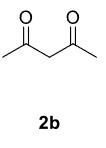	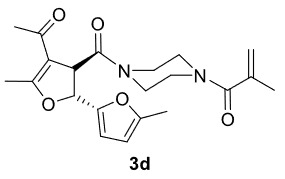	73
5	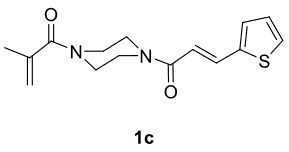	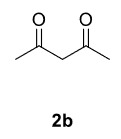	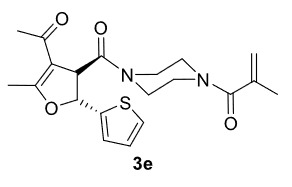	52
6	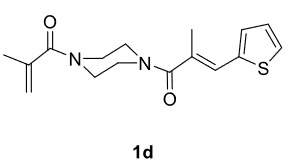	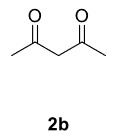	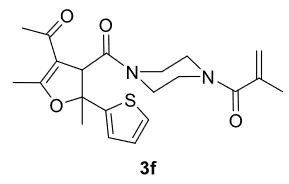	31
7	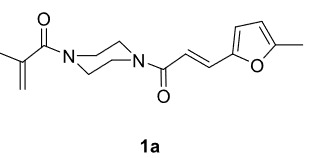	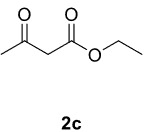	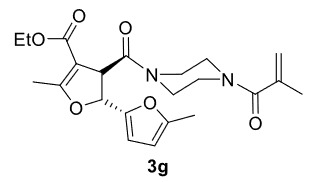	60

a) Isolated yield based on 1,3-dicarbonyl compounds.

**Figure:**
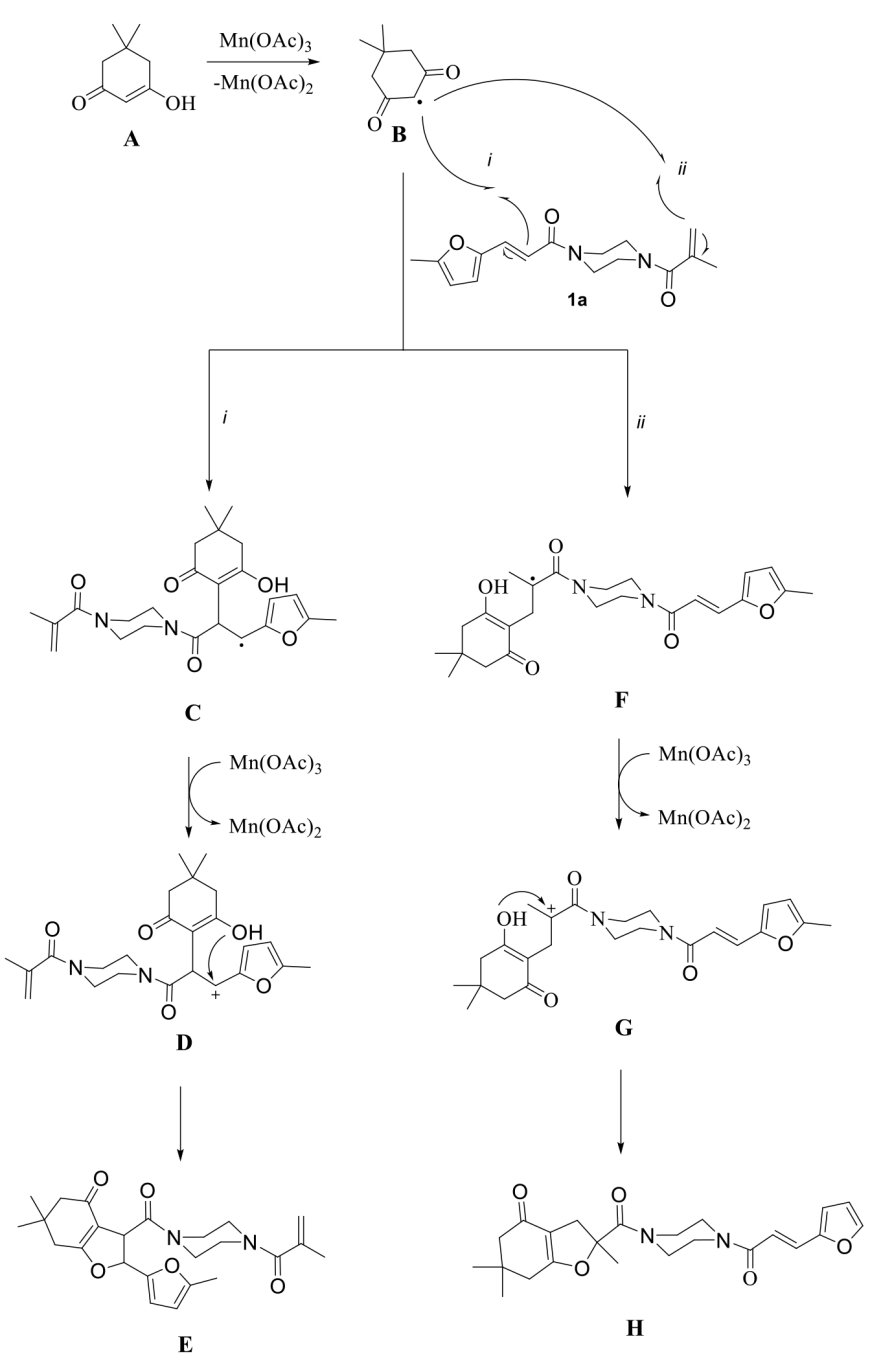
Proposed mechanism of Mn(OAc) _3_ mediated radical cyclization.

The results of the reactions of 1e-hwith 2a are given in Table 2. From the reactions of ally piperazine derivatives (1e-g) with 2a, dihydrofurans 3h (30%), 3i (32%), and 3j (20%) were obtained in low yields. By comparing the yields of 3a (81%) with 3i (32%) and yields of 3c (64%) with 3j (20%) it can be deduced that yields of methacryloyl-piperazine–substituted dihydrofurans are higher than yields of allyl-piperazine–substituted dihydrofurans. Additionally, reactions of 1e-g with 2c formed 3k (25%), 3l (40%), and 3m (20%) in low yields, respectively. Reactions of ally-methacryloyl piperazine (1h) with 2c formed 3n (20%) in low yields.

**Table 2:** Radical cyclizations of 1e-h with 2a and 2c.

Entry	Piperazine	1,3-dicarbonyl	Product	Yield (%) ^a^
1	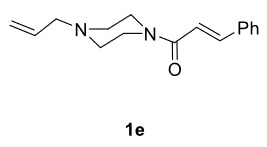	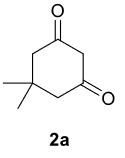	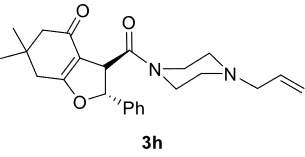	30
2	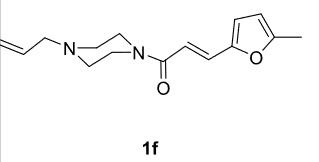	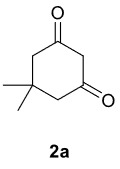	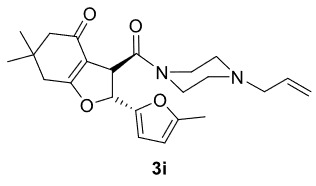	32
3	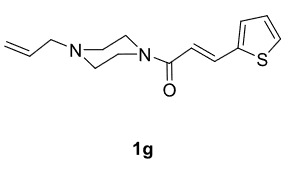	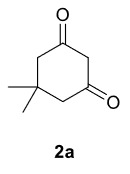	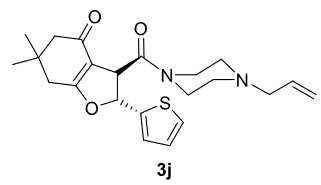	20
4	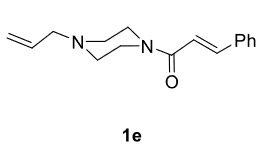	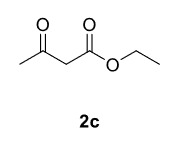	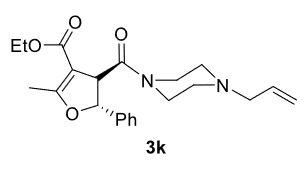	25
5	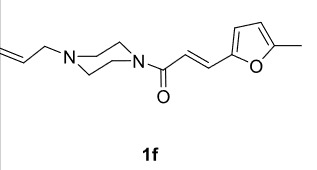	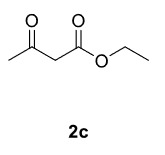	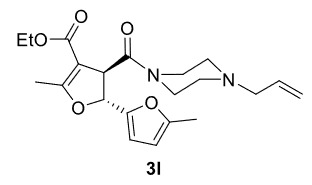	40
6	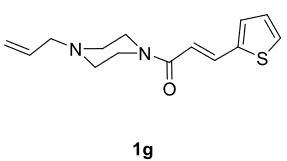	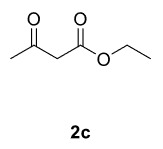	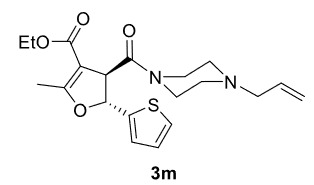	20
7	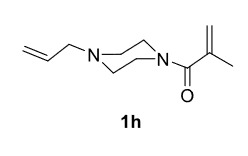	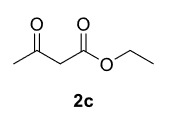	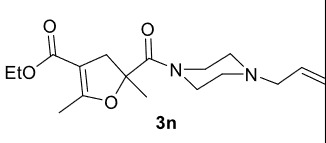	20

a) Isolated yield based on 1,3-dicarbonyl compounds.

Radical cyclizations of unsaturated diacyl and allyl-acyl piperazine compounds (except 1h) occurred regioselectively through 3-arylpropenoyl moiety. However, no cyclization product that formed over ally or methacryloyl moiety was isolated (except 3n). This is due to the fact that radical intermediates formed adjacent to the aromatic rings are much more stable than those formed on allyl or methacryloyl moieties. Similarly, since the radical intermediates formed on methacrylic moiety are much more stable than those formed on the ally group, radical cyclization of 1h and 2c occurred through methacryloyl group to form dihydrofuran-piperazine (3n). The ^1^ H NMR spectra of obtained compounds 3a, 3c-e, and 3g-m show that vicinal dihydrofuran couplings are *J* trans = 5.2–7.6 Hz (in the literature *J* trans = 2.5–7.6 Hz and *J* cis = 8–11 Hz) [45,46,48,49,53–56], thus it was determined that these molecules are trans compounds.

The proposed mechanism for the formation of dihydrofuransis is explained in Figure. According to this mechanism, the enol form of dimedone (A) reacts with Mn(OAc) _3_ , and an alpha carbon radical B is formed, while Mn ^3+^ reduces to Mn2+. There are two possible pathways for this alpha carbon radical to attach to 1a. Radical intermediate C can be formed by following pathway- *i* , and radical intermediate F can be formed by following pathway- *ii* . On pathway- *i* , oxidation of C to carbocation D with Mn(OAc) _3_ and intramolecular cyclization of D forms the product E. Similarly, by following pathway- *ii* , product H is formed. However, on the 1H-NMR spectra of the obtained products, the chemical shifts of two terminal alkene peaks of methacryl group were observed in the range of 5.25–5.00 ppm. Additionally, two vicinal proton peaks of dihydrofurans around 6.00 and 4.51 ppm ( *J* trans= 5.2–6.4 Hz) were observed. According to this information, it was determined that the radical cyclization of 1a-d with 2a-c followed the pathway- *i* , and products 3a-g formed; however, the other possible products (H) were not isolated.

Summarily, reactions of methacryloyl- and 3-arylacryloyl–substituted piperazines (1a-d) with 1,3-dicarbonyls (2a-c) occurs on 3-arylacryloyl sides, regioselectively. However, in reactions of allyl- and acryloyl-substituted (methacryloyl or 3-arylacryloyl) piperazines (1e-h)with 2a or 2c, cyclization occurs at acryloyl moiety, regioselectively, and thus, relevant dihydrofurans (3h-n) were formed. No cyclization occurred on allyl moiety at any reaction.

## 3. Experimental design

### 3.1. Chemicals and equipment

Melting points were determined on a Gallenkamp capillary melting point apparatus (Gallenkamp & Co., London, UK) and IR spectra (ATR, PerkinElmer, Inc. Waltham, MA, USA) were obtained with a Bruker Tensor27 spectrophotometer (Bruker Optics GmbH, Ettlingen, Germany) in the 400–4000 cm ^-1^ range with 2 cm ^-1^ resolutions. The ^1^ H NMR and ^13^ C NMR spectra were recorded on a Varian Mercury-400 high-performance digital FT-NMR and Varian Oxford NMR300 spectrometers (Varian Medical Systems, Inc., Palo Alto, CA, USA). High resolution mass time-of-flight spectra (TOF) were measured on an Agilent 1200/6210 LC/MS spectrophotometer (AgilentTechnologies, Inc., SantaClara, CA, USA). Thin layer chromatography (TLC) was performed on Merck aluminum-packed silica gel plates (Merck&Co., Inc., Kenilworth, NJ, USA). Purification of products was by column chromatography on silica gel (Merck silica gel 60, 40–60 mm), and preparative TLC was on silica gel from Merck (PF _254-366nm_ ) (Kenilworth, NJ, USA). All reagents, 1,3-dicarbonyl compounds, and solvents were commercially purchased. Radical oxidant Mn(OAc) _3_ was synthesized by electrochemical method [57]. Please note that ^1^ H NMR, ^13^ C NMR, and HRMS spectra for all novel compounds can be found as supplementary information.

### 3.2. General synthesis procedure and spectroscopic data of diacyl (3a-g) piperazine-dihydrofuran compounds

A solution of Mn(OAc) _3_ (2mmol, 0.53 g) in 15 mL of glacial acetic acid was heated to 80 °C until dissolved. Then, the solution temperature was set to 65 °C. A solution of the corresponding 1,3-dicarbonyl compound (2a-c) (1mmol) and suitable unsaturated piperazine compound (1a-d)(1.2 mmol) in 2 mL of acetic acid was added to Mn(OAc) _3_ solution. The mixture was stirred, and the disappearance of the dark brown color indicated that the reaction was finished (15–60 min). Water was added, and the crude product was extracted with chloroform (20×3 mL). Combined organic phases were neutralized with saturated NaHCO _3_ solution, dried over anhydrous Na _2_ SO _4_ , and evaporated. The residue was purified with column chromatography (silica gel 60, 40–60 mm) using chloroform–acetone (85:15) as eluent. All compounds were further purified by preparative TLC (PF _254-366nm_ ) before spectroscopic analyses.

***Trans* -3-(4-Methacryloylpiperazine-1-carbonyl)-6,6-dimethyl-2-(5-methylfuran-2-yl)-3,5,6,7-tetrahydrobenzofuran-4(2H)-one (3a)**

It was obtained as a yellow oil; yield: 81% (0.345 g); IR (ATR) υmax 3000, 2957, 2926, 1720 (C=O), 1618 (C=O), 1606 (C=C), 1197, 1022, 748 (arom. CH) cm ^-1^ ; ^1^ H NMR (400 MHz, CDCl _3_ ) d (ppm): 6.30 (1H, d, *J* = 3.2 Hz, arom. CH), 6.00 (1H, d, *J* = 5.6 Hz, H-2), 5.96 (1H, d, *J* = 3.2 Hz, arom. CH), 5.21 (1H, s, H _olef._ ), 5.04 (1H, s, H _olef._ ), 4.51 (1H, d, *J* = 5.6 Hz, H-3), 4.07-3.27 (8H, m), 2.43 (1H, d, *J* = 17.6 Hz), 2.33 (1H, d, *J* = 17.6 Hz), 2.33 (1H, d, *J* = 16.4 Hz), 2.28 (3H, s, -CH _3_ ), 2.19 (1H, d, *J* = 16.4 Hz), 1.95 (3H, s, -CH _3_ ), 1.13 (3H, s, -CH _3_ ), 1.11 (3H, s, -CH _3_ ); ^13^ C NMR(100 MHz, CDCl _3_ ) d (ppm):193.9 (C=O), 177.3 (C=C, C-7a), 171.3 (C=O), 169.9 (C=O), 154.0, 148.6, 140.0 (C=C), 115.9 (C=C), 112.3, 111.1,106.7(C=C, C-3a), 83.6 (C-2), 51.1, 46.6, 45.0 (C-3), 42.2, 37.9, 34.4, 28.9 (-CH _3_ ), 28.2 (-CH _3_ ), 20.4 (-CH _3_ ), 13.6 (-CH _3_ );HRMS (ESI)(m/z) Calcd for C _24_ H _30_ N _2_ O _5_ 427.22275 found 427.22472 (M+H) ^+^ .

**3-(4-Methacryloylpiperazine-1-carbonyl)-2,6,6-trimethyl-2-(5-methylfuran-2-yl)-3,5,6,7-tetrahydrobenzofuran-4(2H)-one (3b)**

It was obtained as a yellow oil; yield: 50% (0.220 g); IR (ATR) υmax3093, 2956, 2925, 1721 (C=O), 1635 (C=O), 1610 (C=C), 1194, 1020, 728 (arom. CH) cm ^-1^ ; ^1^ H NMR (400 MHz, CDCl _3_ ) d (ppm):6.24 (1H, d, *J* = 3.2Hz, arom. CH), 5.94 (1H, d, *J* = 3.2 Hz), 5.22 (1H, s, H _olef_ ), 5.03 (1H, s, H _olef_ ), 4.58 (1H, s, H-3), 3.79-3.52 (8H, m), 2.40 (1H, d, *J* = 17.6 Hz), 2.35 (1H, d, *J* = 17.6 Hz), 2.32 (1H, d, *J* = 16.0 Hz), 2.20 (1H, d , *J* = 16.0 Hz), 2.30 (3H, s, -CH _3_ ), 1.94 (3H, s, -CH _3_ ), 1.68 (3H, s, -CH _3_ ), 1.20 (3H, s, -CH _3_ ), 1.11 (3H, s, -CH _3_ ); ^13^ C NMR(100 MHz, CDCl _3_ ) d (ppm): 194.1 (C=O), 175.4 (C=C, C-7a), 171.3 (C=O), 167.8 (C=O), 153.2, 152.8, 139.8 (C=C), 116.1 (C=C), 112.9, 112.5, 106.5 (C=C, C-3a), 88.1 (C-2), 50.7, 49.0 (C-3), 45.9, 42.5, 37.8, 34.6, 28.6 (-CH _3_ ), 28.5 (-CH _3_ ), 21.1 (-CH _3_ ), 20.4 (-CH _3_ ), 13.6 (-CH _3_ );HRMS (ESI)(m/z) Calcd forC _25_ H _32_ N _2_ O _5_ 441.23840 found 441.23896 (M+H) ^+^ .

***Trans* -3-(4-Methacryloylpiperazine-1-carbonyl)-6,6-dimethyl-2-(thiophen-2-yl)-3,5,6,7-tetrahydrobenzofuran-4(2H)-one (3c)**

It was obtained as a yellow oil; yield: 64% (0.274 g); IR (ATR) υmax3085, 2956, 2930, 1732 (C=O), 1639 (C=O), 1615 (C=C), 1197, 1026, 726 (arom. CH)cm ^-1^ ; ^1^ H NMR (400 MHz, CDCl _3_ ) d (ppm): 7.34 (1H, d, *J* = 5.2 Hz, arom. CH), 7.06 (1H, d, *J* = 3.6 Hz, arom. CH), 7.00 (1H, dd, *J* = 5.2, 3.6 Hz, arom. CH), 6.32 (1H, d, *J* = 5.2 Hz, H-2), 5.21 (1H, s, H _olef_ ), 5.04 (1H, s, H _olef_ ), 4.36 (1H, d, *J* = 5.2 Hz, H-3), 4.03-3.28 (8H, m), 2.47 (1H, d, *J* = 17.6 Hz), 2.35 (1H, d, *J* = 17.6 Hz), 2.32 (1H, d, *J* = 16.4 Hz), 2.20 (1H, d, *J* = 16.4 Hz), 1.94 (3H, s, -CH _3_ ), 1.14 (3H, s, -CH _3_ ), 1.12 (3H, s, -CH _3_ ); ^13^ C NMR(100 MHz, CDCl _3_ ) d (ppm): 193.9 (C=O), 177.0 (C=C, C-7a), 171.2 (C=O), 169.8 (C=O), 141.9, 140.0 (C=C), 115.9 (C=C), 127.1, 126.6, 126.3, 111.9 (C=C, C-3a), 86.1 (C-2), 51.1, 50.0 (C-3), 46.5, 42.2, 37.9, 34.4, 28.9 (-CH _3_ ), 28.1 (-CH _3_ ), 20.4 (-CH _3_ ); HRMS (ESI) (m/z) Calcd for C _23_ H _28_ N _2_ O _4_ S429.18425 found 429.18588 (M + H) ^+^ .

***Trans* -1-(4-(4-Acetyl-5,5’-dimethyl-2,3-dihydro-[2,2’-bifuran]-3-carbonyl)piperazin-1-yl)-2-methylprop-2-en-1-one (3d)**

It was obtained as a yellow oil; yield: 73% (0.282 g); IR (ATR) υmax3009, 2956, 2930, 1732 (C=O), 1652 (C=O), 1612 (C=C), 1193, 1020, 746 (arom. CH)cm ^-1^ ; ^1^ H NMR (400 MHz, CDCl _3_ ) d (ppm): 6.29 (1H, d, *J* = 3.2 Hz, arom. CH), 5.95 (1H, d, *J* = 3.2 Hz, arom. CH), 5.52 (1H, d, *J* = 6.4 Hz, H-2), 5.20 (1H, s, H _olef_ ), 5.02 (1H, s, H _olef_ ), 4.69 (1H, d, *J* = 6.4 Hz, H-3), 3.80-3.66 (8H, m), 2.31 (3H, s, -CH _3_ ), 2.30 (3H, s, -CH _3_ ), 2.29 (3H, s, -CH _3_ ), 1.93 (3H, s, -CH _3_ ); ^13^ C NMR(100 MHz, CDCl _3_ ) d (ppm): 192.8 (C=O), 171.3 (C=C, C-7a), 171.2 (C=O), 167.6 (C=O), 155.5, 153.8, 140.0 (C=C), 116.7 (C=C), 115.9, 110.9, 106.7 (C=C, C-3a), 79.9 (C-2), 49.1 (C-3), 46.2, 42.7, 28.8 (-CH _3_ ), 20.4 (-CH _3_ ), 15.6 (-CH _3_ ), 13.6 (-CH _3_ );HRMS (ESI)(m/z) Calcd for C _21_ H _26_ N _2_ O _5_ 387.19145 found 387.19223 (M+H) ^+^ .

***Trans* -1-(4-(4-Acetyl-5-methyl-2-(thiophen-2-yl)-2,3-dihydrofuran-3-carbonyl)piperazin-1-yl)-2-methylprop-2-en-1-one (3e)**

It was obtained as a yellow oil; yield: 52% (0.202g); IR (ATR) υmax3085, 2998, 2917, 1714 (C=O), 1639 (C=O), 1610 (C=C), 1194, 1020, 724 (arom. CH)cm ^-1^ ; ^1^ H NMR (400 MHz, CDCl _3_ ) d (ppm): 7.33 (1H, d, *J* = 5.2 Hz, arom. CH), 7.05 (1H, d, *J* = 3.6 Hz, arom. CH), 7.00 (1H, dd, *J* = 5.2, 3.6 Hz, arom. CH), 5.86 (1H, d, *J* = 6.4 Hz, H-2), 5.20 (1H, s, H _olef_ ), 5.02 (1H, s, H _olef_ ), 4.54 (1H, d, *J* = 6.4 Hz, H-3), 3.81-3.39 (8H, m), 2.34 (3H, s, -CH _3_ ), 2.33 (3H, s, -CH _3_ ), 1.93 (3H, s, -CH _3_ ); ^13^ C NMR(100 MHz, CDCl _3_ ) d (ppm): 192.8 (C=O), 171.3 (C=C, C-7a), 171.1 (C=O), 167.6 (C=O), 144.6, 140.0 (C=C), 127.1, 126.4, 126.1, 116.6 (C=C), 115.9 (C=C, C-3a), 82.6 (C-2), 53.5 (C-3), 46.5, 42.4, 28.9 (-CH _3_ ), 20.4 (-CH _3_ ), 15.6 (-CH _3_ )192.9HRMS (ESI)(m/z) Calcd for C _20_ H _24_ N _2_ O _4_ S389.15295found 389.15460 (M+H) ^+^ .

**1-(4-(4-Acetyl-2,5-dimethyl-2-(thiophen-2-yl)-2,3-dihydrofuran-3-carbonyl)piperazin-1-yl)-2-methylprop-2-en-1-one (3f)**

It was obtained as a yellow oil; yield: 31% (0.125 g); IR (ATR) υmax3117, 2953, 2918, 1734 (C=O), 1648 (C=O), 1615 (C=C), 1191, 1022, 724 (arom. CH)cm ^-1^ ; ^1^ H NMR (400 MHz, CDCl _3_ ) d (ppm): 7.27 (1H, d, *J* = 6.4 Hz, arom. CH), 6.97-6.95 (2H, m, arom. CH), 5.22 (1H, s, H _olef_ ), 5.04 (1H, s, H _olef_ ), 4.54 (1H, s, H-3), 3.68-3.32 (8H, m), 2.35 (3H,s, -CH _3_ ), 2.30 (3H,s, -CH _3_ ), 1.94 (3H,s, -CH _3_ ), 1.74 (3H,s, -CH _3_ ); ^13^ C NMR(100 MHz, CDCl _3_ ) d (ppm): 192.9 (C=O), 171.3 (C=C, C-7a), 168.9 (C=O), 166.5 (C=C, C-3a), 149.6, 139.9 (C=C), 126.9, 125.0, 123.0, 116.3 (C=C), 116.0 (C=C, C-3a), 86.4 (C-2), 56.6 (C-3), 46.3, 42.6, 28.7 (-CH _3_ ), 24.0 (-CH _3_ ), 20.4 (-CH _3_ ), 15.4 (-CH _3_ );HRMS (ESI)(m/z) Calcd for C _21_ H _26_ N _2_ O _4_ S403.16860 found 403.16968 (M+H) ^+^ .

***Trans* -Ethyl 3-(4-methacryloylpiperazine-1-carbonyl)-5,5’-dimethyl-2,3-dihydro-[2,2’-bifuran]-4-carboxylate (3g)**

It was obtained as a yellow oil; yield: 60% (0.250 g); IR (ATR) υmax3080, 2980, 2922, 1701 (C=O), 1641 (C=O), 1619 (C=C), 1194, 1020, 730 (arom. CH)cm ^-1^ ; ^1^ H NMR (400 MHz, CDCl _3_ ) d (ppm): 6.30 (1H, d, *J* = 3.2 Hz, arom. CH), 5.95 (1H, d, *J* = 3.2 Hz, arom. CH), 5.55 (1H, d, *J* = 5.6 Hz, H-2), 5.21 (1H, s, H _olef_ ), 5.02 (1H, s, H _olef_ ), 4.67 (1H, d, *J* = 5.6 Hz, H-3),4.16 (2H, q, *J* = 7.2 Hz, -OCH2CH _3_ ), 3.57-3.40 (8H, m), 2.30 (3H, s, -CH _3_ ), 2.25 (3H, s, -CH _3_ ), 1.93 (3H, s, -CH _3_ ), 1.27 (3H, t, *J* = 7.2 Hz, -OCH2CH _3_ ); ^13^ C NMR(100 MHz, CDCl _3_ ) d (ppm): 171.3 (C=O), 171.2 (C=C, C-7a), 168.8 (C=O), 165.0 (C=O), 153.7, 148.6, 139.9 (C=C), 116.0 (C=C), 110.8, 106.7, 103.8 (C=C, C-3a), 80.2 (C-2), 59.9, 48.7 (C-3), 46.4, 42.6, 20.4 (-CH _3_ ), 14.5 (-CH _3_ ), 14.4 (-CH _3_ ), 13.6 (-CH _3_ );HRMS (ESI)(m/z) Calcd for C _22_ H _28_ N _2_ O _6_ 417.20201 found 417.20397 (M+H) ^+^ .

## 3.3. General synthesis procedure and spectroscopic data of alkyl-acyl (3h-n) piperazines–dihydrofuran compounds

A solution of Mn(OAc) _3_ (2mmol, 0.53 g ) in 15 mL of glacial acetic acid was heated to 80 °C until dissolved. Then, the solution temperature was set to 65 °C. A solution of the corresponding 1,3-dicarbonyl compound (2a or 2c) (1mmol) and the suitable unsaturated piperazine compound (1e-h) (1.2 mmol) in 2 mL of acetic acid was added to Mn(OAc) _3_ solution. The mixture was stirred, and the disappearance of the dark brown color indicated that the reaction was finished (15–60 min). Water was added, and the crude product was extracted with chloroform (20 × 3 mL). Combined organic phases were neutralized with saturated NaHCO _3_ solution, dried over anhydrous Na _2_ SO _4_ , and evaporated. The residue was purified with column chromatography (silica gel 60, 40–60 mm) using chloroform–acetone (85:15) as eluent. All compounds were further purified by preparative TLC (PF _254-366nm_ ) before spectroscopic analyses.

***Trans* -3-(4-Allylpiperazine-1-carbonyl)-6,6-dimethyl-2-phenyl-3,5,6,7-tetrahydrobenzofuran-4(2H)-one (3h)**

It was obtained as a yellow oil; yield: 30% (0.118 g); IR (ATR) υmax3067, 2961, 2868, 1730 (C=O), 1632 (C=O), 1612 (C=C), 1197, 1020, 754, 701 (arom. CH)cm ^-1^ ; ^1^ H NMR (400 MHz, CDCl _3_ ) d (ppm):7.39-7.31 (3H, m, arom. CH), 7.24-7.22 (2H, m, arom. CH), 6.04 (1H, d, *J* = 5.6 Hz, H-2), 5.83 (1H, ddt, *J* = 16.8, 10, 6.4 Hz, H _olef._ ), 5.18 (1H, dd, *J* = 16.8, 1.2 Hz, H _olef._ ), 5.14 (1H, dd, *J* = 10.0, 1.2 Hz, H _olef._ ), 4.23 (1H, d, *J* = 5.6 Hz, H-3), 3.96-3.84 (2H, m), 3.50-3.39 (2H, m), 3.00 (2H, d, *J* = 6.4 Hz), 2.56-2.44 (4H, m), 2.31 (2H, d, *J* = 16.0 Hz), 2.18 (2H, d, *J* = 16.0 Hz), 1.27 (3H, s, -CH _3_ ), 1.16 (3H, s, -CH _3_ ); ^13^ C NMR(100 MHz, CDCl _3_ ) d (ppm):193.7(C=O), 177.4 (C=C, C-7a), 169.9 (C=O), 139.9, 134.3 (C=C), 129.0, 128.8, 125.5, 118.5 (C=C), 112.2 (C=C, C-3a), 90.5 (C-2), 61.4,53.1, 52.6, 51.1, 49.7, 46.2, 42.4 (C-3), 34.3, 28.9 (-CH _3_ ), 28.2(-CH _3_ );HRMS (ESI)(m/z) Calcd for C _24_ H _30_ N _2_ O _3_ 395.23292 found 395.23361 (M+H) ^+^ .

***Trans* -3-(4-Allylpiperazine-1-carbonyl)-6,6-dimethyl-2-(5-methylfuran-2-yl)-3,5,6,7-tetrahydrobenzofuran-4(2H)-one (3i)**

It was obtained as a yellow oil; yield: 32% (0.127 g); IR (ATR) υmax30171, 2961, 2930, 1732 (C=O), 1641 (C=O), 1617 (C=C), 1197, 1002, 734 (arom. CH)cm ^-1^ ; ^1^ H NMR (400 MHz, CDCl _3_ ) d (ppm):6.28 (1H, d, *J* = 3.2 Hz, arom. CH), 5.94 (1H, d, *J* = 6.0 Hz, H-2), 5.93 (1H, d, *J* = 3.2 Hz, arom. CH), 5.83 (1H, ddt, *J* = 16.8, 10, 6.8 Hz, H _olef_ ), 5.18 (1H, dd, *J* = 16.8, 1.6 Hz, H _olef_ ), 5.14 (1H, dd, *J* = 10.0, 1.6 Hz, H _olef_ ), 4.49 (1H, d, *J* = 6.0 Hz, H-3), 3.99-3.89 (2H, m), 3.49-3.37 (2H, m), 2.99 (2H, d, *J* = 6.8 Hz), 2.59-2.49 (4H, m), 2.40 (1H, d, *J* = 17.6 Hz), 2.30 (1H, d, *J* = 17.6 Hz), 2.29 (1H, d, *J* = 16.0 Hz), 2.26 (3H, s, -CH _3_ ), 2.17 (1H, d, *J* = 16.0 Hz), 1.12 (3H, s, -CH _3_ ), 1.08 (3H, s, -CH _3_ ); ^13^ C NMR(100 MHz, CDCl _3_ ) d (ppm):193.8 (C=O), 176.8 (C=C, C-7a), 169.5 (C=O), 153.8, 148.9, 134.3 (C=C), 118.5 (C=C), 112.5, 110.8, 106.6 (C=C, C-3a), 83.5 (C-2), 61.4, 53.0, 52.5, 51.1, 46.2, 45.5, 42.3 (C-3), 37.8, 34.4, 28.9(-CH _3_ ), 28.1(-CH _3_ ), 13.6(-CH _3_ );HRMS (ESI)(m/z) Calcd for C _23_ H _30_ N _2_ O _4_ 399.22783 found 399.22924 (M+H) ^+^ .

***Trans* -3-(4-Allylpiperazine-1-carbonyl)-6,6-dimethyl-2-(thiophen-2-yl)-3,5,6,7-tetrahydrobenzofuran-4(2H)-one (3j)**

It was obtained as a yellow oil; yield: 20% (0.080 g); IR (ATR) υmax3076, 2955, 2924, 1731 (C=O), 1628 (C=O), 1617 (C=C), 1137, 1000, 750 (arom. CH)cm ^-1^ ; ^1^ H NMR (400 MHz, CDCl _3_ ) d (ppm): 7.32 (1H, d, *J* = 5.2 Hz, arom. CH), 7.06 (1H, d, *J* = 3.6 Hz, arom. CH), 6.99 (1H, dd, *J* = 5.2, 3.6 Hz, arom. CH), 6.28 (1H, d, *J* = 5.2 Hz, H-2), 5.85 (1H,ddt, *J* =16.8, 10.0,6.8 Hz, H _olef_ ), 5.20 (1H, dd, *J* = 16.8, 1.6 Hz, H _olef_ ), 5.18 (1H, dd, *J* = 10.0, 1.6 Hz, H _olef_ ), 4.37(1H,d, *J* =5.2 Hz, H-3), 4.00-3.96 (2H,m), 3.54-3.42 (2H, m), 3.04 (2H,d, *J* =6.8 Hz), 2.58 (4H, s), 2.46 (1H, d, *J* = 17.6 Hz), 2.33 (1H, d, *J* = 17.6 Hz), 2.32 (1H, d, *J* = 16.0 Hz), 2.20 (1H, d, *J* = 16.0 Hz), 1.14 (3H, s, -CH _3_ ), 1.11 (3H, s, -CH _3_ ); ^13^ C NMR(100 MHz, CDCl _3_ ) d (ppm):193.8 (C=O), 176.7 (C=C, C-7a), 169.3 (C=O), 143.7, 133.9 (C=C), 128.4, 127.0, 126.4, 116.6 (C=C), 112.1 (C=C, C-3a), 86.1 (C-2), 61.3, 53.0, 52.5, 51.1, 49.8, 46.1, 42.2 (C-3), 37.9, 34.4, 28.9 (-CH _3_ ), 28.1 (-CH _3_ );HRMS (ESI)(m/z) Calcd for C _22_ H _28_ N _2_ O _3_ S400.23319 found 401.1894 (M+H) ^+^ .

***Trans* -Ethyl 4-(4-allylpiperazine-1-carbonyl)-2-methyl-5-phenyl-4,5-dihydrofuran-3-carboxylate (3k)**

It was obtained as a yellow oil; yield: 25% (0.096g); IR (ATR) υmax3080, 2978, 2930, 1701 (C=O), 1659 (C=O), 1619 (C=C), 1203, 969, 730, 692 (arom. CH) cm ^-1^ ; ^1^ H NMR (400 MHz, CDCl _3_ ) d (ppm): 7.40-7.27 (5H, m, arom. CH), 5.81 (1H, ddt, *J* =17.2, 10.4, 6.4 Hz, H _olef_ ), 5.61 (1H, d, *J* = 7.2 Hz, H-2), 5.17 (1H, dd, *J* = 16.8, 1.6 Hz, H _olef_ ), 5.15 (1H, dd, *J* = 10.4, 1.6 Hz, H _olef_ ), 4.33 (1H, d, *J* = 7.2 Hz, H-3), 4.13 (2H, q, *J* = 7.2 Hz, -OCH2CH _3_ ), 3.84-3.79 (1H, m), 3.64-3.58 (1H, m), 3.48-3.34(2H, m), 2.97 (2H, d, *J* = 6.8 Hz), 2.53-2.36 (4H, m), 2.35 (3H, s, -CH _3_ ), 1.24 (3H, t, *J* = 7.2 Hz, -OCH2CH _3_ ); ^13^ C NMR(100 MHz, CDCl _3_ ) d (ppm):171.2 (C=O), 169.6 (C=C, C-7a), 164.9 (C=O), 140.3, 134.4 (C=C), 128.9, 128.6, 125.4, 118.4 (C=C), 103.6 (C=C, C-3a), 87.3 (C-2), 61.4, 59.7, 53.1, 52.9, 52.8, 42.4 (C-3), 14.4 (-CH _3_ ), 14.3 (-CH _3_ );HRMS (ESI)(m/z) Calcd for C _22_ H _28_ N _2_ O _4_ 385.21218 found 385.21370 (M+H) ^+^ .

***Trans* -Ethyl 3-(4-allylpiperazine-1-carbonyl)-5,5’-dimethyl-2,3-dihydro-[2,2’-bifuran]-4-carboxylate (3l)**

It was obtained as a yellow oil; yield: 40% (0.155 g); IR (ATR) υmax3067, 2982, 2907, 1708 (C=O), 1663 (C=O), 1626 (C=C), 1199, 1100, 732 (arom. CH) cm ^-1^ ; ^1^ H NMR (400 MHz, CDCl _3_ ) d (ppm): 6.26 (1H, d, *J* = 3.2 Hz, arom. CH), 5.92 (1H, d, *J* = 3.2 Hz, arom. CH), 5.80 (1H, ddt, *J* = 16.8, 10.0, 6.4 Hz, H _olef_ ), 5.48 (1H, d, *J* = 7.6 Hz, H-2), 5.14 (1H, dd, *J* = 16.8, 1.6 Hz, H _olef_ ), 5.13 (1H, dd, *J* = 10.0, 1.6 Hz, H _olef_ ), 4.62 (1H, d, *J* = 7.6 Hz, H-3), 4.12 (2H, q, *J* = 7.2 Hz, -OCH2CH _3_ ), 3.76-3.71 (1H, m), 3.58-3.49 (2H, m), 3.45-3.40 (1H, m), 2.94 (2H, d, *J* = 6.4 Hz), 2.49-2.13 (4H, m), 2.27 (3H, s, -CH _3_ ), 2.23 (3H, s, -CH _3_ ), 1.22 (3H, t, *J* = 7.2 Hz, -OCH2CH _3_ ); ^13^ C NMR(100 MHz, CDCl _3_ ) d (ppm):170.7 (C=O), 168.7 (C=C, C-7a), 164.9 (C=O), 153.5, 148.9, 134.4 (C=C), 118.4 (C=C), 110.5, 106.5, 103.8 (C=C, C-3a), 80.2 (C-2), 61.4, 59.7, 53.0, 52.6, 48.6, 46.0, 42.3 (C-3), 14.4(-CH _3_ ), 14.3(-CH _3_ ), 13.6(-CH _3_ );HRMS (ESI)(m/z) Calcd for C _21_ H _28_ N _2_ O _5_ 389.20710 found 389.20877 (M+H) ^+^ .

***Trans* -Ethyl 4-(4-allylpiperazine-1-carbonyl)-2-methyl-5-(thiophen-2-yl)-4,5-dihydrofuran-3-carboxylate (3m)**

It was obtained as a yellow oil; yield: 20% (0.078 g); IR (ATR) υmax3075, 2978, 2923, 1701 (C=O), 1652 (C=O), 1628 (C=C), 1197, 1040, 728 (arom. CH) cm ^-1^ ; ^1^ H NMR (400 MHz, CDCl _3_ ) d (ppm): 7.32 (1H, dd, *J* = 5.2 Hz, arom. CH), 7.05 (1H, d, *J* = 3.2 Hz, arom. CH), 6.98 (1H, dd, *J* = 5.2, 3.2 Hz, arom. CH), 5.84 (1H, d, *J* = 6.8 Hz, H-2), 5.81 (1H, ddt, *J* = 16.8, 10.0, 6.4 Hz, H _olef_ ), 5.18 (1H, dd, *J* = 16.8, 1.6 Hz, H _olef_ ), 5.15 (1H, dd, *J* = 10.0, 1.6 Hz, H _olef_ ), 4.48 (1H,d, *J* = 6.8 Hz, H-3), 4.15 (2H, q, *J* = 7.2 Hz, -OCH2CH _3_ ), 3.83-3.45 (4H, m), 2.98 (2H, d, *J* = 6.4 Hz), 2.52-2.18 (4H, m), 2.29 (3H, s, -CH _3_ ), 1.25 (3H, t, *J* = 7.2 Hz, -OCH2CH _3_ ); ^13^ C NMR(100 MHz, CDCl _3_ ) d (ppm):170.6 (C=O), 168.9 (C=C, C-7a), 164.8 (C=O), 142.5, 134.3 (C=C), 126.9, 126.1, 125.8, 118.5 (C=C), 103.7 (C=C, C-3a), 82.9 (C-2), 61.4, 59.8, 53.1, 53.0, 52.7, 46.1, 42.4 (C-3), 14.5 (-CH _3_ ), 14.4 (-CH _3_ );HRMS (ESI)(m/z) Calcd for C _20_ H _26_ N _2_ O _4_ S391.16860 found 391.16985 (M+H) ^+^ .

**Ethyl 5-(4-allylpiperazine-1-carbonyl)-2,5-dimethyl-4,5-dihydrofuran-3-carboxylate(3n)**

It was obtained as a yellow oil; yield: 20% (0.065 g); IR (ATR) υmax3076, 2983, 2930, 1701 (C=O), 1657 (C=O), 1630 (C=C), 1190, 1040cm ^-1^ ; ^1^ H NMR (400 MHz, CDCl _3_ ) d (ppm): 5.84 (1H, ddt, *J* = 16.8, 10, 6.8 Hz, H _olef_ ), 5.20 (1H, dd, *J* = 16.8, 1.6 Hz, H _olef_ ), 5.17 (1H, dd, *J* = 10.0, 1.6 Hz, H _olef_ ), 4.15 (2H, q, *J* = 7.2 Hz, -OCH2CH _3_ ), 3.81-3.62 (4H, m), 3.58 (1H, d, *J* = 15.2 Hz, Ha-3), 3.00 (2H, d, *J* = 6.8 Hz), 2.70 (1H, d, *J* = 15.2 Hz, Hb-3), 2.44 (4H, m), 2.18 (3H, s, -CH _3_ ), 1.55 (3H, s, -CH _3_ ), 1.26 (3H, t, *J* = 7.2 Hz, -OCH2CH _3_ ); ^13^ C NMR(100 MHz, CDCl _3_ ) d (ppm):170.1 (C=O), 165.7 (C=C, C-7a), 164.8 (C=O), 134.3 (C=C), 118.5 (C=C), 102.1 (C=C, C-3a), 88.4 (C-2), 61.5, 59.6, 53.1, 52.8, 46.2, 43.2, 41.1 (C-3), 26.0(-CH _3_ ), 14.3(-CH _3_ ), 14.1(-CH _3_ );HRMS (ESI)(m/z) Calcd for C17H26N2O4323.19653 found 323.19805 (M+H) ^+^ .

## 4. Conclusion

Summarily, novel piperazines containing dihydrofuran compounds (3a-n) were synthesized by the Mn(OAc) _3_ mediated radical cyclization from unsaturated diacyl (1a-d) and alkyl-acyl (1e-h) piperazine compounds with 1,3-dicarbonyls (2a-c) in low to high yields for the first time. All compounds were characterized by ^1^ HNMR, ^13^ C NMR, HRMS, and FTIR spectroscopy.
